# Alum and Squalene-Oil-in-Water Emulsion Enhance the Titer and Avidity of Anti-Aβ Antibodies Induced by Multimeric Protein Antigen (1–11)E2, Preserving the Igg1-Skewed Isotype Distribution

**DOI:** 10.1371/journal.pone.0101474

**Published:** 2014-07-01

**Authors:** Francesca Mantile, Maria Trovato, Andrea Santoni, Pasquale Barba, Simone Ottonello, Piergiuseppe De Berardinis, Antonella Prisco

**Affiliations:** 1 Institute of Genetics and Biophysics, CNR, Napoli, Italy; 2 Institute of Protein Biochemistry, CNR, Napoli, Italy; 3 Department of Life Sciences, University of Parma, Parma, Italy; Instituto Butantan, Brazil

## Abstract

The development of active immunotherapy for Alzheimer's disease (AD) requires the identification of immunogens that can ensure a high titer antibody response toward Aβ, while minimizing the risks of adverse reactions. Multimeric protein (1–11)E2 induces a robust and persistent antibody response to Aβ in mice, when formulated in Freund's adjuvant. The goal of this translational study was to evaluate the immunogenicity of (1–11)E2 formulated in alum (Alhydrogel 2%), or in a squalene oil-in-water emulsion (AddaVax), or without adjuvant. A IgG1-skewed isotype distribution was observed for the anti-Aβ antibodies generated in mice immunized with either the non-adjuvanted or the adjuvanted vaccine, indicating that (1–11)E2 induces a Th2-like response in all tested conditions. Both Alhydrogel 2% and AddaVax enhanced the titer and avidity of the anti-Aβ response elicited by (1–11)E2. We conclude that (1–11)E2 is a promising candidate for anti-Aβ immunization protocols that include alum or squalene-oil-in-water emulsion, or no adjuvant.

## Introduction

Active and passive immunization studies performed in transgenic mouse models of Alzheimer's disease have demonstrated that antibodies against Aβ are able to reduce plaques and improve cognition (reviewed in [1]–[3]). In mouse models of AD and in clinical trials, induction of high titer anti-Aβ antibodies correlated with the reduction in brain Aβ [4]–[7]. Individuals with the highest titers of anti-Aβ antibodies displayed the most pronounced depletion of plaques [4]. The development of a Th1-type response was found to correlate with an adverse inflammatory reaction [8].

The development of a vaccine for the prevention or therapy of Alzheimer's Disease faces two challenges, namely, overcoming the low immunogenicity of the Aβ peptide and avoiding detrimental inflammatory reactions in the brain [1]. We have previously described a multimeric protein antigen for the induction of an antibody response to Aβ that consists of a domain of the bacterial protein E2 able to self-assemble into a particle consisting of 60 monomers [9]. Peptides encompassing the 1–11 and 2–6 amino acid positions of Aβ were displayed as N-terminal fusions on the surface of the E2 particles [9]. E2-based vaccines induced a fast-rising, robust and persistent antibody response to Aβ in all vaccinated mice, with a higher titer of anti-Aβ antibodies in the case of the vaccine harboring the 1–11 Aβ peptide, i.e. vaccine (1–11)E2. Balb/c mice receiving (1–11)E2 formulated in Complete Freund's Adjuvant (CFA) developed immune memory to Aβ after a single dose, as demonstrated by the fact that a booster dose, administered 6 months after the first dose, induced a very high serum concentration of anti-Aβ antibodies (above 1 mg/ml) [9]–[11]. Vaccination with (1–11)E2 in Complete Freund's Adjuvant - Incomplete Freund's Adjuvant (CFA-IFA) polarizes the immune response toward the production of the anti-inflammatory cytokine Interleukin-4 and does not induce a T cell response to Aβ [9].

As CFA-IFA is a very strong and reactogenic adjuvant not suitable for human use, in this study we analyze the magnitude, kinetics, isotype, avidity and specificity towards distinct Aβ species of the anti- Aβ response elicited by vaccine (1–11)E2 formulated in alum (Alhydrogel 2%), in a squalene-based oil-in-water emulsion (AddaVax), or without adjuvant; human vaccines containing Alhydrogel or oil-in-water adjuvants have been licensed. Compared to our previous study [9] a detoxified (1–11)E2 preparation was employed in this work in order to eliminate potential confounding effects caused by lipopolysaccharide (LPS), a typical contaminant of proteins produced in *E. coli* and a strong activator of innate immunity.

## Materials and Methods

### Ethics statement

Protocols involving mice have been approved by the Ethics Committee of the Ministero della Salute, Dipartimento della Sanità Pubblica Veterinaria, della Sicurezza Alimentare e degli organi collegiali per la Tutela della Salute Direzione Generale della Sanità Animale e dei Farmaci Veterinari, and conform to the provisions of the Declaration of Helsinky and Italian National guidelines for animal use in research.

### Animals

Female (C57BL/6 x C3H)F1 mice (henceforth named B6C3/F1 mice), were obtained from Charles River Laboratory, Italy, and immunized at 8 weeks of age.

Hemizygous female B6C3-Tg(APPswe, PSEN1dE9)85Dbo/Mmjax (004462) mice (henceforth named APP PSEN1 mice), were obtained from the Mutant Mouse Regional Resource Centers and immunized at 8 weeks of age.

### Antigen preparation

(1–11)E2 was produced and characterized as previously described [9] and stored at −80°C. To obtain LPS-free (1–11)E2, protein samples were purified from lipopolysaccharide (LPS) by phase separation with TritonX-114 (Sigma) [12], followed by detergent removal with Thermo Scientific Pierce Detergent Removal Resin (Thermo Scientific). Samples were tested for LPS using the Limulus Amebocyte Lysate (LAL) Assay (Lonza) according to the manufacturer' s instructions. Alhydrogel 2% (alum) and AddaVax (squalene-oil-in-water) were purchased from InvivoGen, California, USA. AddaVax is based on nano-emulsification of two components: Sorbitan trioleate (0.5% w/v) in squalene oil (5% v/v); Tween 80 (0.5% w/v) in sodium citrate buffer (10 mM, pH 6.5). The nano-emulsion is produced using a microfluidizer and filtered through a 0.22-µm filter to remove large droplets and sterilize the final product, the particle size is ∼160 nm, as described in the product information provided by InvivoGen, Version # 11K09-MM.

On the day of immunization, (1–11)E2 was mixed with adjuvant or PBS following manufacturer's protocols. In particular, (1–11)E2 particles were mixed 1∶1 with AddaVax by inversion just prior to use, whereas in the case of Alhydrogel 2%, (1–11)E2 particles were mixed 1∶1 with Alhydrogel 2%, and incubated at room temperature for 1 hour on a rotator to allow antigen adsorption.

In order to verify adsorption, at the end of the incubation period the antigen-Alhydrogel 2% was centrifuged at 5000 g for 4 minutes at room temperature, and the amount of unbound protein in the supernatant was determined by measuring the protein concentration using the Coomassie dye binding method (Bradford assay). No unbound protein was detected in the supernatant; the absorbance of the antigen-Alhydrogel 2% supernatant was identical to the absorbance of the supernatant of a negative control consisting of PBS-Alhydrogel 2%.

### Immunizations

Mice were immunized subcutaneously with 130 µg of (1–11)E2 particles, mixed 1∶1 with adjuvant or PBS, in a final volume of 200 µl per dose. The amount of Alhydrogel 2% adjuvant per dose utilized corresponds to the higher value of the range recommended by the manufacturer for mouse experimentation. In experiment 1, B6C3/F1 mice received 3 doses of LPS-free vaccine, at day 0, 21 and 210. In experiment 2, B6C3/F1 mice received 3 doses of vaccine, at day 0, 15 and 238. In experiment 3, APP PSEN1 mice received 3 doses of vaccine at day 0, 15 and 30; each dose consisted of 130 µg of (1–11)E2 particles, mixed 1∶1 with Alhydrogel 2%.

### Enzyme-linked immunosorbent assay (ELISA)

ELISA assays were performed as previously described [9], [10], [13]. Wells of a 96-well Nunc Immunoplate were coated with 100 µl of 5 µg/ml streptavidin at 37°C over night until complete evaporation. Wells were blocked with 0.5% bovine serum albumin in 20 mM TrisHCl pH 7.3, and 120 mM NaCl, incubated with 50 ng biotinylated peptide, incubated with mouse sera diluted in 0.25% bovine serum albumin, 20 mM TrisHCl pH 7.3, 0.5 M NaCl, 0.05% Tween 20, and detected with anti-mouse IgG peroxidase conjugate (SIGMA A-2554), anti-mouse IgG1 peroxidase conjugate (BD Pharmingen 559626) and anti-mouse IgG2a peroxidase conjugate (BD Pharmingen 553391). All incubations were carried out for 1 h at 37°C, and after each step wells were washed twice with Elisa wash buffer (EWB) (20 mM TrisHCl pH 7.3, 130 mM NaCl, 0.05% Tween 20) and once with Tris buffered saline (TBS) (20 mM TrisHCl pH 7.3, 0.5 M NaCl). Wells were incubated for 45 min at room temperature with 0.4 mgml^−1^ O-phenylenediamine dihydrochloride dissolved in 30 mM citric acid, 70 mM Na_2_HPO_4_, 0.8 mM H_2_O_2_. Absorbance was read at 492 nm, after blocking color development with 0.8 M sulfuric acid.

Each serum was tested against synthetic peptide 1–11 of Aβ. Synthetic peptide 23–29 of Aβ was used as a negative control. Peptides were biotinylated on a lysine residue added to the C-terminus; biotin was attached to the ε-amino group of the lysine side chain. Titer of a serum was defined as the dilution yielding an absorbance value equal to twofold the background value obtained against the negative control.

When indicated, sera samples were also tested against aggregated synthetic Aβ42. To obtain aggregated Aβ42, 1 mg of Aβ42 (Innovagen SP-BA42-5) was dissolved in 830 µl of water and vortexed to generate a uniform suspension; an aliquot of 100 µl of 10 x PBS was added, and the suspension was vortexed again; 50 µl of NaHPO4 1 M pH 7.5 and 20 µl of NaCl 5M were added, and the solution was vortexed and incubated at 37°C over night. Wells of ELISA plates were coated with 1 µg per well of aggregated Aβ42 in phosphate-buffered saline at 4 C for 16 h.

### Titration of IgG1 and IgG2a isotype antibodies

IgG1 and IgG2a isotype antibodies were detected respectively with secondary anti-mouse IgG1 peroxidase conjugate BD Pharmingen 559626 and secondary anti-mouse IgG2a peroxidase conjugate BD Pharmingen 553391. In order to demostrate that the secondary antibodies give comparable signals at the same concentration of primary antibody, titration curves with IgG1 isotype antibody Bam-10 (Sigma-Aldrich A3981) and IgG2a isotype antibody NAB-228 (Sigma-Aldrich A8354) were performed. The concentration of the purified Bam-10 and NAB-228 antibodies was determinated with the Coomassie dye binding method based on the Bradford assay, utilizing the Bio-Rad Protein Assay. Wells of a 96-well Nunc Immunoplate were incubated at 4°C over night with 50 µl of a serial dilution of either Bam-10 or NAB-228, starting from the concentration of 5.76 ng/ml. Wells were blocked with 0.5% bovine serum albumin in 20 mM TrisHCl pH 7.3, and 120 mM NaCl, and detected with anti-mouse IgG1 peroxidase conjugate (BD Pharmingen 559626) and anti-mouse IgG2a peroxidase conjugate (BD Pharmingen 553391). The titration curves that demonstrate that the secondary antibodies give the same signal at the same concentration of primary antibody are shown in (figure S1).

### Anti-Aβ avidity determination

The avidity index of sera for synthetic peptide 1–11 of Aβ and for pre-aggregated Aβ42, expressed as the percentage avidity, was obtained from the ratio of ELISA absorbance values in the presence or absence of Urea 6 M as described in [14]. Sera were plated in quadruplicate. After washes in EWB and TBS as described above, half of the wells were incubated for 10 minutes at 37°C with 100 µl of Urea 6 M, while the other wells were incubated with PBS. The avidity was calculated as the ratio of absorbance measured at 490 nm in urea-treated and PBS-treated wells (A_UREA_/A_PBS_)x 100. The serum dilutions were chosen to yield, in the PBS-treated wells, a value of absorbance equal to 0.5+/−0.1, in the linear phase of the titration curve [14].

### Immuno-dot blot analysis of (1–11)E2 antisera reactivity against different Aβ species

Distinct Aβ species were generated from the water-soluble depsipeptide derivative of Aβ42 (Aβ42-depsi; RP10017-1, GenScript) as described previously [15], [16].

Unaggregated, predominantly monomeric Aβ42 was prepared by dissolving Aβ42-depsi in PBS (pH 7.4) at a 450 µg/ml concentration, followed by centrifugation at 4°C for 40 min. at 18000×g, quantitation with the Qubit Protein Assay Kit (Molecular Probes) on a Qubit 2.0 Fluorometer, and dilution to the final working concentration (7.5 or 30 µg/ml) with PBS. The linear dynamic range of the Qubit Protein Assay was checked on serial dilutions of monomeric Aβ42 peptide, and the lack of effect of the Aβ conformation on the Qubit Protein Assay quantification was verified on Aβ monomers, oligomers and protofibrils obtained from the same Aβ42 aliquot, and that had not undergone a centrifugation step (A. S., unpublished data).

Aβ oligomers were generated by incubating the dissolved Aβ42-depsipeptide at 4°C for 12–16 hours, followed by centrifugation and quantitation as above, and dilution of the supernatant to the final working concentration with PBS. For Aβ42 protofibril production, the Aβ42-depsipeptide was dissolved at a 1350 µg/ml concentration in 2 mM NaOH, diluted to 675 µg/ml with 100 mM HCl and incubated for 24–48 hours at 37°C. After centrifugation and quantitation as above, the supernatant was diluted with PBS to the same working concentrations utilized for Aβ monomers and oligomers. For Aβ42 fibril production, the Aβ42-depsipeptide was dissolved and diluted as described above for the protofibrils and incubated for 9 days at 37°C. Fibril samples were not centrifuged and after quantitation they were directly diluted to the final working concentration with PBS. Aβ oligomer and (proto)fibril formation, as well as the absence of fibrillar aggregates in monomeric and oligomeric Aβ preparations, were verified by atomic force microscopy with a Nanoscope III microscope (Digital Instruments) as described [17]. For dot blot analysis, 15 ng or 60 ng of the various Aβ42 species, in a volume of 2 µl, were hand-spotted onto 0.2 µm nitrocellulose membranes (BioRad) pre-wetted with TBS (20 mM Tris-HCl, pH 7.5, 0.8% NaCl). After blocking for 2 hours at room temperature with Odyssey Blocking Buffer (LI-COR), membranes were washed three times for 10 min with TBST (20 mM Tris-HCl, pH 7.5, 0.8% NaCl, 0.2% Tween20), followed by incubation for 1 h at room temperature with individual (1–11)E2 antisera in Odyssey Blocking Buffer. Membranes were then washed three times with TBST and incubated for 1 h at RT with a labeled anti-mouse immunoglobulin antibody (IRDye 680, LI-COR; diluted 1∶5000). After an additional three washes with TBST, blots were dried and analyzed with an Odyssey infrared fluorescence imager (LI-COR) at different laser intensities.

Individual pools of (1–11)E2 antisera obtained with the use of different adjuvant or without adjuvant, were tested at different dilutions, including the anti-Aβ (1–11) end-point titers derived from ELISA.

### Statistical analysis

The two-tailed Student's t test was performed to determine statistical significance of observed differences. Data on samples taken from the same mouse at different time-points were analyzed with the paired test, different treatment groups were analyzed with the unpaired test with unequal variances. The p-value was calculated with Microsoft Excel:Mac 2004.

## Results

### Non-adjuvanted, LPS-free (1–11)E2 is immunogenic and induces immunological memory along with an IgG1-skewed immune response

**Figure 1:**
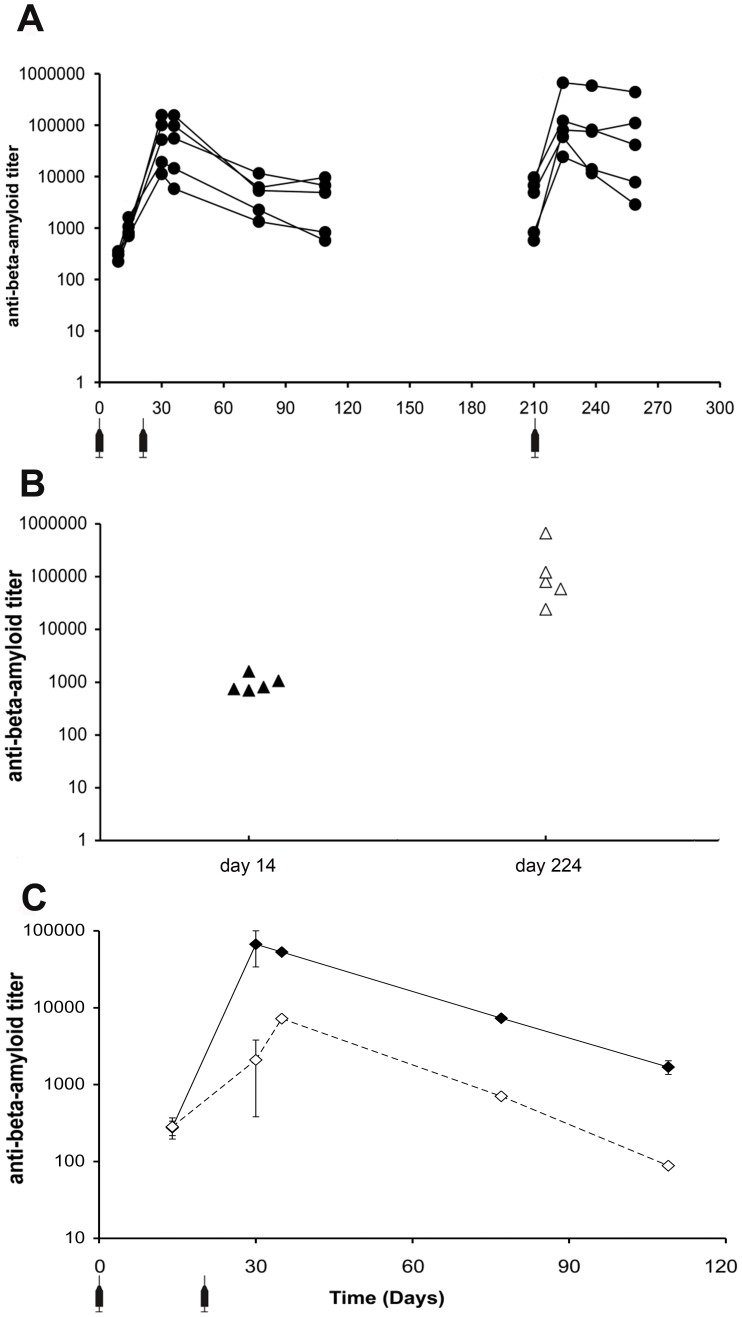
Titer and isotype of anti-Aβ antibodies produced in mice immunized with LPS-free (1–11)E2 without adjuvant. **panel a** The graph shows the anti-Aβ antibody IgG titer measured on the sera of five B6C3/F1 mice immunized with LPS-free (1–11)E2, without adjuvant, at the indicated time-points. The antibody response elicited by the dose administered at day 210 is higher than the primary response, indicating the development of immunological memory. All immunized mice produced anti-Aβ antibodies after the first dose, and exhibited a recall response. **panel b** The graph reports the anti-Aβ IgG titer in the sera of individual B6C3/F1 mice, 14 days after the first dose of vaccine, and 14 days after the recall dose (day 224). All immunized mice produced anti-Aβ antibodies after the first dose, and exhibited a recall response. **panel c** The graph reports the anti-Aβ titer of IgG1 (closed diamonds) and IgG2a (open diamonds) isotype antibodies in the pooled sera of 5 B6C3/F1 mice immunized with LPS-free (1–11)E2 at day 0 and 21. The error bars represent the standard deviation, calculated from extreme alternative fit lines. From day 21 onwards, the IgG1 titer is consistently higher than the IgG2a titer.

We have previously reported that (1–11)E2, formulated in CFA-IFA, is highly immunogenic [9]. As CFA-IFA is a very strong and reactogenic adjuvant that is not suitable for human use, we set out to profile the immunogenicity of (1–11)E2 in the absence of adjuvants. (1–11)E2 is produced in *E. coli*, and chromatography-purified preparations are invariably contaminated by traces of LPS, an activator of innate immunity that can have an adjuvant effect. In this experiment we have utilized LPS-free (1–11)E2, obtained from a further round of purification, as described in ‘Materials and methods’. A group of five B6C3/F1 mice were immunized subcutaneously with LPS-free (1–11)E2, diluted in PBS, at day 0, 21 and 210. Sera were collected at different time-points over a 10 months period. In all sera, we observed a peak of anti-Aβ antibodies at day 30 (figure 1 a). The recall dose, administered at day 210, elicited a recognizable memory response, characterized by a quicker increase in anti-Aβ antibody titers and a more persistent response (figure 1 a). As shown in figure 1 a, all immunized mice started to produce measurable concentrations of anti-Aβ antibodies 14 days after the first injection and all of them developed immune memory, as revealed by the high anti-Aβ titer observed at day 224, i.e. 14 days after the recall dose (figure 1 b).

Within an antibody response, the IgG1/IgG2a ratio is considered a correlate of the polarization of the T cell response, with Th1 T-cell responses favoring the production of IgG2a and Th2 T-cell responses favoring the production of IgG1 [18]. We analyzed the titer of IgG1 and IgG2a isotype anti-Aβ antibodies obtained after immunization of B6C3/F1 mice with non-adjuvanted LPS-free (1–11)E2 (figure 1 c). On the pooled sera from 5 immunized mice we found that the IgG response to Aβ induced by non-adjuvanted LPS-free (1–11)E2 is characterized by a marked prevalence of the IgG1 isotype over the IgG2a isotype (figure 1 c), suggesting that the ability to induce a Th2 T cell response, previously observed for CFA-IFA adjuvanted (1–11)E2 [9], is an intrinsic quality of LPS-free (1–11)E2. In order to rule out the effect of outliers on the values determined in pooled sera, we also analyzed individual sera, confirming the prevalence of IgG1 (figure S2 a).

### Alhydrogel and AddaVax enhance the titer and avidity of the anti-Aβ response induced by LPS-free (1–11)E2, preserving the IgG1-skewed isotype distribution

We then set out to evaluate the immunogenicity of (1–11)E2, formulated in two different adjuvants, namely Alhydrogel 2% (alum), and AddaVax, a squalene oil-in-water emulsion.

**Figure 2:**
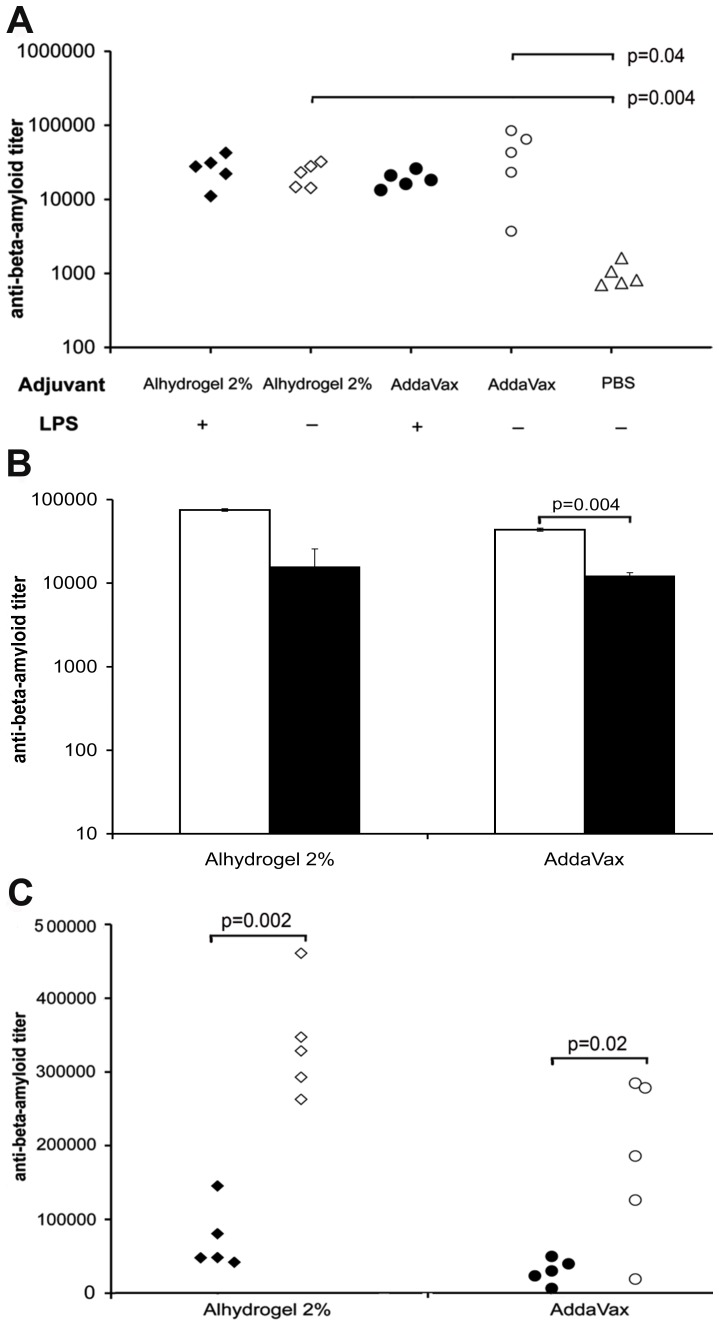
Titer and isotype of anti-Aβ antibodies induced by (1–11)E2 formulated in Alhydrogel 2% or AddaVax. **panel a** The plot reports the anti-Aβ antibody titer at day 14 of B6C3/F1 mice that have received one injection of (1–11)E2, formulated in Alhydrogel 2% (diamonds), AddaVax (circles), or without adjuvant (triangles). Each symbol represents a mouse. Some mice were immunized with LPS-free (1–11)E2 (open symbols) while other mice were immunized with (1–11)E2 obtained with the standard purification protocol (closed symbols). With either adjuvant, the presence of LPS contamination in the (1–11)E2 preparation has no statistically significant effect on the anti-Aβ titer. Both Alhydrogel 2% and AddaVax significantly enhanced the anti-Aβ response induced by LPS-free (1–11)E2, with no significant difference in the anti-Aβ IgG titer obtained with Alhydrogel 2%, or AddaVax. **panel b** The histograms show the anti-Aβ titer of IgG1 (white bars) and IgG2a (black bars) isotype antibodies in the pooled sera of B6C3/F1 mice (n = 5 in each treatment group) vaccinated with LPS-free (1–11)E2, formulated in the indicated adjuvants. Sera were collected at day 109. The error bars represent the standard deviation, calculated from extreme alternative fit lines. IgG1 antibodies were more abundant than IgG2a in both the Alhydrogel 2% and the AddaVax group. **panel c** The graph shows the anti-Aβ (1–11) IgG titer in the sera of B6C3/F1 mice immunized with (1–11)E2 formulated in either Alhydrogel 2% or AddaVax. Mice received 3 doses of adjuvanted vaccine, at day 0, 15 and 238. Sera were collected before the day 238 recall dose (black symbols) and 14 days after the day 238 recall dose (open symbols). A memory response was observed both in the Alhydrogel 2% and in the AddaVax group.

As our previous study on (1–11)E2 immunogenicity [9] was performed with chromatographically purified (1–11)E2 preparations containing trace amounts of LPS, we evaluated the potential effect of this trace contaminant on immunogenicity by comparing anti-Aβ antibody titers in mice that received one dose of detoxified or non-detoxified (1–11)E2 antigen formulated in either Alhydrogel 2% or Addavax. The amount of LPS in the non-detoxified (1–11)E2 was 4330 EU/ml, wheras in the detoxified (1–11)E2, henceforth named LPS-free (1–11)E2, it was below the detection limit of the LAL test. The chromatographically purified preparation causes IFN-gamma production in peripheral lymphocytes from non-immunized mice (FM and AP, unpublished data). However, no statistically significant effect of LPS (4330 EU/ml) on anti-Aβ titers at day 14 was observed with Alhydrogel 2% or AddaVax adjuvanted (1–11)E2 (figure 2 a). As further shown in figure 2a, both adjuvants significantly enhanced the anti-Aβ response induced by LPS-free (1–11)E2.

Neither Alhydrogel 2%, nor AddaVax altered the IgG subclass distribution observed with the non-adjuvanted vaccine; regardless of the specific adjuvant employed, IgG1 were more abundant than IgG2a immunoglobulins (figure 2 b), indicating that LPS-free (1–11)E2 elicits a Th2-like response with both Alhydrogel 2% and AddaVax. To rule out a potential effect of outliers on the IgG isotype abundance values obtained from pooled sera, we analyzed all available individual sera, confirming the prevalence of IgG1 (figure S2 b, c). An IgG1 skewed anti-Aβ response was also observed in APP PSEN1 mice immunized with 3 doses of (1–11)E2 formulated in Alhydrogel 2% (figure S2 d).

Both Alhydrogel 2% and AddaVax treated mice developed immunological memory against (1–11)E2, as evidenced by a rapid rise in the anti-Aβ titer 14 days after a recall dose of the adjuvanted vaccine administered 6 months after the initial immunization (days 0–15) (figure 2 c).

**Figure 3:**
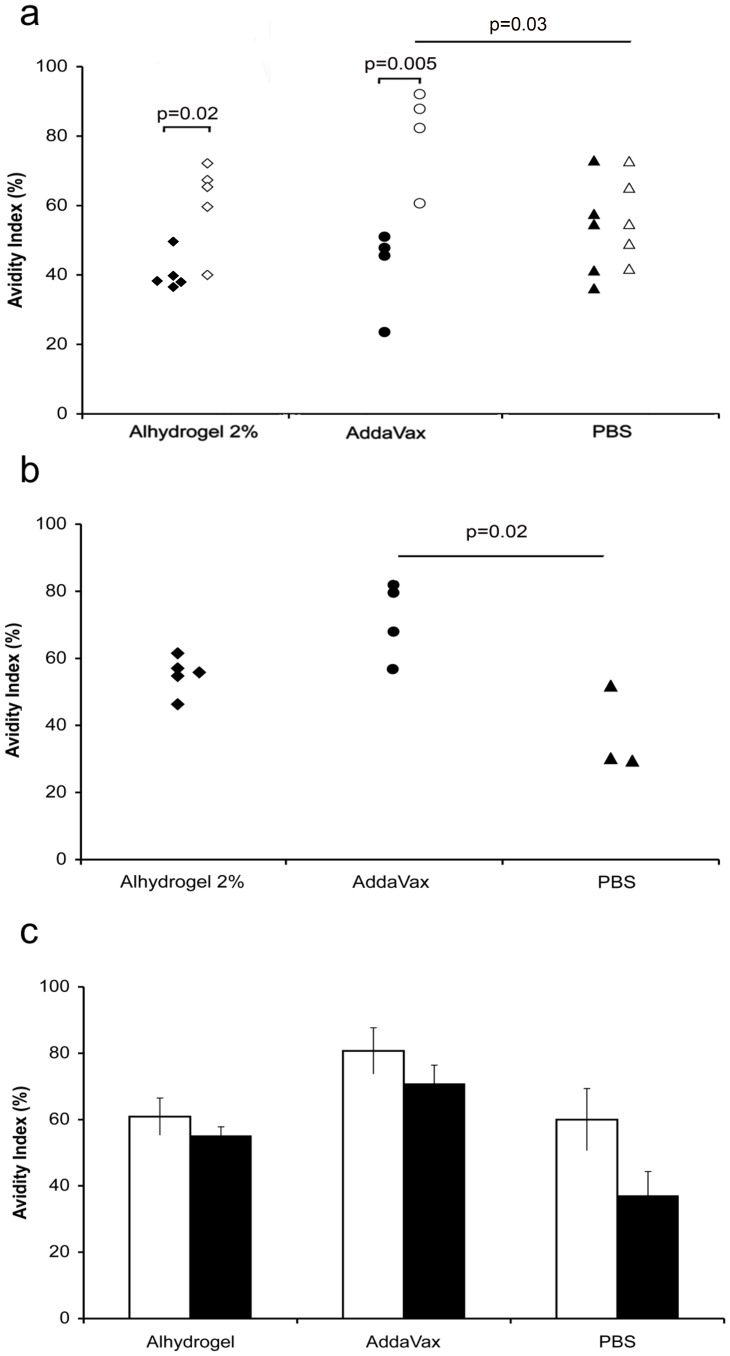
Avidity of anti-Aβ antibodies induced by (1–11)E2 formulated in Alhydrogel 2%, AddaVax or PBS. **panel a** The plot shows the avidity index against Aβ (1–11) of mouse sera collected at day 9 (black symbols) and day 109 (open symbols) from B6C3/F1 mice immunized with LPS-free (1–11)E2 in the indicated adjuvants. Each symbol represents a mouse serum. The avidity index of sera from mice immunized with the adjuvanted vaccine increased significantly from day 9 to day 109, while no increase was observed in the sera of mice immunized without adjuvant. **panel b** The plot shows the avidity index against pre-aggregated Aβ42 of mouse sera collected day 109 (open symbols) from B6C3/F1 mice immunized with LPS-free (1–11)E2 in the indicated adjuvants. Each symbol represents a mouse serum. The avidity index of sera from mice immunized with the AddaVax adjuvanted vaccine is significantly higher than the avidity index of sera from mice immunized with vaccine in PBS. **panel c** The histogram shows the average avidity index against Aβ (1–11) (open bars) and pre-aggregated Aβ42 (black bars) of mouse sera (day 109) from B6C3/F1 mice immunized with LPS-free (1–11)E2 in Alhydrogel 2% (n = 5 mice), AddaVax (n = 4), PBS (n = 3). The error bars represent the standard error of the mean. The avidity against Aβ (1–11) and Aβ42 is very similar in the groups that received the adjuvanted vaccine.

In Alhydrogel 2% and AddaVax treated mice we observed a significant increase in the avidity index of anti-Aβ (1–11) antibodies from day 9 to day 109, whereas no significant maturation of avidity was observed in mice that had received non-adjuvanted LPS-free (1–11)E2 (figure 3 a).

All sera also recognized the full lenght antigen, pre-aggregated Aβ42 (figure 3 b). Both adjuvants enhanced the avidity of day 109 sera against pre-aggregated Aβ42 (figure 3 b). Average avidity against pre-aggregated Aβ42 was very similar to the average avidity measured against the synthetic (1–11) peptide in day 109 sera from mice that had received the adjuvanted vaccine (figure 3 c).

### Immune reactivity profiles of (1–11)E2 antisera against different Aβ species

**Figure 4:**
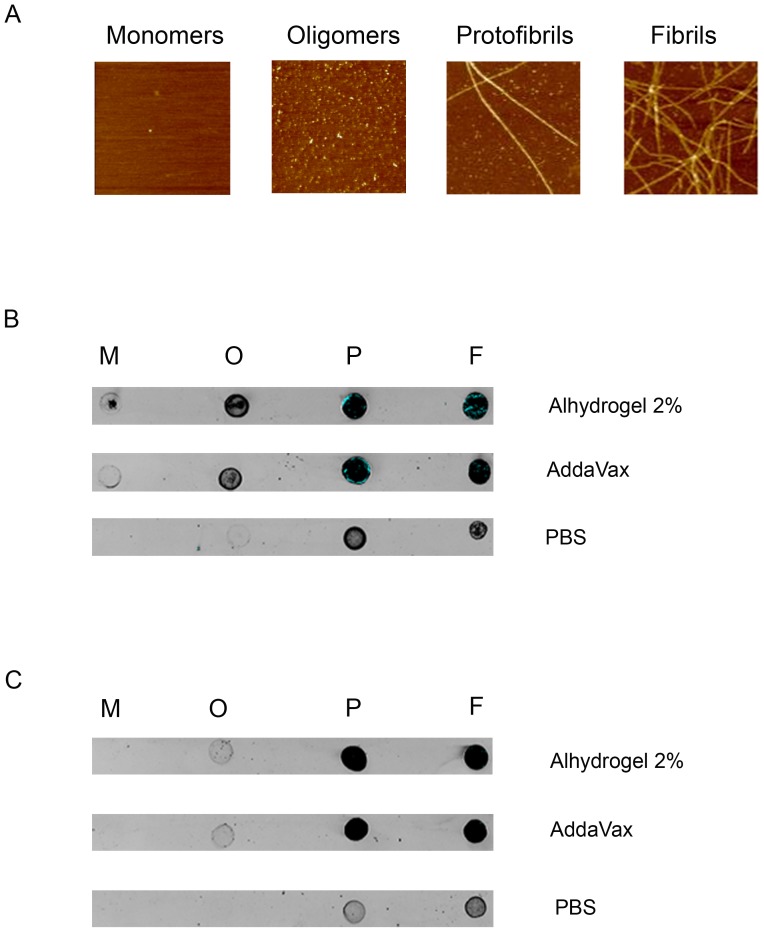
Recognition of distinct Aβ species by sera from mice immunized with differently adjuvanted formulations of the LPS-free (1–11)E2 antigen. **panel a** Representative 1 µm×1 µm atomic force microscopy images of the indicated Aβ42 species (see ‘Materials and methods’ for details). **panel b** Immuno-dot blot analysis conducted on 60 ng each of the various Aβ42 species (*M*, monomers; *O*, oligomers; *P*, protofibrils; *F*, fibrils) at a 1∶5000 dilution of pooled (day 77) sera from B6C3/F1 mice immunized with Alhydrogel- or Addavax-adjuvanted LPS-free (1–11)E2, or with the same antigen without adjuvant (PBS) as indicated. **panel c** Immuno-dot blot analysis conducted on 15 ng each of the various Aβ42 species at a 1∶5000 dilution of pooled (day 77) sera from B6C3/F1 mice immunized with Alhydrogel- or Addavax-adjuvanted LPS-free (1–11)E2, or with the same antigen without adjuvant (PBS).

Next, we examined the immune reactivity profile of (1–11)E2 antisera by dot blot analysis of monomeric, oligomeric, protofibrillar and fibrillar Aβ (figure 4 a). As shown in figure 4 b, sera from B6C3/F1 mice immunized with LPS-free (1–11)E2 preferentially recognize aggregated Aβ species, namely oligomers, protofibrils and fibrils. When lower amounts of Aβ were spotted on the membrane (15 instead of 60 ng/dot as for the analysis reported in panel b), the oligomer signal faded, whereas the proto(fibril) signal was still clearly detectable (figure 4 c), indicating a higher titer (or avidity) of antibodies recognizing higher-order Aβ aggregates (fibril and protofibrils) compared to anti-Aβ oligomer antibodies. In fact, both Aβ fibrils and protofibrils, but not Aβ oligomers, could be detected at (1–11)E2 antisera dilutions as high as 1∶140,000 (Alhydrogel adjuvant) or 1∶80,000 (Addavax adjuvant) (figure S4). Monomeric Aβ was the least recognized species.

The immune reactivity pattern of (1–11)E2 antibodies generated in the presence of the two different adjuvants was similar to that of the unadjuvanted antigen, the main difference being a generally lower anti-Aβ titer in mice immunized with the unadjuvanted antigen (figure S4).

## Discussion

The goal of this study was to evaluate the ability of multimeric protein antigen (1–11)E2, formulated either in Alhydrogel 2%, or AddaVax, or in the absence of adjuvant, to induce anti-Aβ antibody. Human vaccines containing Alhydrogel (alum) have been licensed. AddaVax is a squalene-based oil-in-water nano-emulsion with a formulation similar to MF59, an adjuvant that has been licensed in Europe for adjuvanted flu vaccines. LPS-free (1–11)E2 was utilized in this study, on the assumption that LPS, a trace contaminant of chromatography purified (1–11)E2 preparation and a strong activator of innate immunity, might affect the antibody response induced by the vaccine.

Adsorption of (1–11)E2 to Alhydrogel 2% was very efficient, as no free antigen was detected. The isoelectric point of (1–11)E2 is 6.4 and the isoelectric point of Alhydrogel 2% is ∼11 [19], therefore, in PBS, electrostatic effects can favor adsorption of (1–11)E2 to Alhydrogel. The amount of Alhydrogel per dose utilized in this study matches the higher limit of the range recommended by the manufacturer for mouse experimentation; the efficacy of lower amounts of Alhydrogel will be tested in future studies.

The presence of LPS in the (1–11)E2 preparation utilized for the experiment in figure 2 did not affect the anti-Aβ titer under our immunization conditions. Remarkably, the LPS-containing (1–11)E2 induced an IgG1 bias in vivo (FM and AP, unpublished data), despite the fact that in cultured mouse lymphocytes the same amount of LPS (4330 EU/ml) strongly induces IFN-gamma production (FM and AP, unpublished data).

We observed that, in the absence of adjuvant, LPS-free (1–11)E2 is immunogenic and induces anti-Aβ antibodies with a prevalence of the IgG1 isotype, suggestive of a Th2 polarization. Both Alhydrogel 2% and AddaVax enhance the titer of the anti-Aβ response induced by LPS-free (1–11)E2, without changing the isotype of the response.

In this study, a large titer variation was seen between individual mice, despite the fact that the mice were genetically identical, and age and sex matched. This variability may reflect stochastic differences in the number of vaccine-specific lymphocytes that are activated in individual animals upon vaccination. The titer of the antibody response is considered a crucial parameter to obtain therapeutic efficacy, therefore highly variable responses among individuals may implicate that some vaccinees do not to achieve protective antibody titers. The protective anti-Aβ titer for the prevention of Alzheimer's disease has not been identified yet. In a previous study, we observed a reduction of Aβ plaques in mice with anti-Aβ titers above 1∶1000 at the time of sacrifice. From the experiments reported here, it appears that two doses of non-adjuvanted, LPS-free (1–11)E2 vaccine administered at day 0 and 21 are not sufficient to ensure persistence of anti-Aβ titers above 1∶1000 in all individuals for longer than 3 months (figure 1 a). In contrast, when the vaccine is formulated in Alhydrogel or Addavax, the anti-Aβ titer is maintained above 1∶15000 in all individuals for at least 3 months (figure S3).

In mice that received the adjuvanted vaccine, the avidity of anti-Aβ (1–11) antibodies increased significantly from day 9 to day 109. Avidity for the full-lenght antigen Aβ42 was higher in mice immunized with adjuvant (p = 0.02 for AddaVax). All antisera preferentially recognized the most aggregated Aβ forms, namely oligomers, protofibrils and fibrils. In particular, protofibrils and fibrils were the best recognized species, being recognized by all antisera also at the lowest amount of target tested (15 ng/dot) (figure 4 c), and at the highest dilutions of sera (figure S4).

The differences we have observed in the immuno-reactivity pattern of sera from mice immunized with the adjuvanted and the non-adjuvanted antigen can be attributed to the ability of adjuvants to enhance the titer of the anti-Aβ response, in fact when all sera were analyzed at a dilution equal to the respective titer measured by ELISA against Aβ (1–11), the immune reactivity profiles appeared very similar, as only binding to protofibrils and fibrils could be detected (figure S4).

In the Alzheimer's brain, Aβ monomers, oligomers and fibrils exist in a complex equilibrium. In an environment that contains different Aβ species, antibody binding to each Aβ species depends on the respective binding avidities, on antibody concentration and on the concentration of the different Aβ species. The antibody titers in the central nervous system are known to be much lower than antibody titers in plasma; for instance, the CNS level of an anti-Aβ monoclonal was found to be 0.1% of the plasma levels [20]. It has been speculated that anti-Aβ antibodies binding Aβ monomers in plasma may modulate Aβ CNS levels by acting as a “peripheral sink” and drive efflux of Aβ from the central nervous system [21], [22], however an alternative scenario is that the main effect of antibody binding to monomeric Aβ in blood is the prolongation of Aβ half-life, with the formation of a highly stable complex, that cannot mediate additional effects on Aβ through binding [23]. Activity in acute reversal of the conditional fear conditioning (CFC) has been reported to correlate inversely with recognition of monomeric Aβ by monoclonal antibodies against the N-terminus of beta-amyloid [24]. Aβ oligomers and protofibrils have been reported to be toxic, albeit there is still a lack of a common, agreed-upon experimental description of the toxic Aβ oligomer [25]. In mice, the ability of monoclonal antibodies to bind fibrillar Aβ is associated with efficacy in passive immunotherapy treatments begun following onset of plaque deposition ([26], [20], reviewed in [27]). In this context, it is interesting that the pooled sera of mice immunized with (1–11)E2 Alhydrogel recognized Aβ protofibrils and fibrils at a 1∶140000 serum dilution, and that antisera were able to recognize soluble Aβ aggregates, namely oligomers and soluble protofibrils, that are considered the most toxic Aβ species. The relatively lower avidity for monomeric Aβ displayed by (1–11)E2 antisera could be an advantage, as it would reduce antibody binding to Aβ monomers in plasma, and therefore increase CNS exposure.

Overall, we conclude that Alhydrogel 2% and AddaVax enhance the immunogenicity of (1–11)E2 without changing the immunoglobulin G1 (IgG1) dominated profile of the humoral immune responses to Aβ induced by the non- adjuvanted antigen. By comparison, we have previously found that an N-terminal Aβ epitope displayed on a different carrier, namely a filamentous bacteriophage, elicits antibodies with a prevalence of IgG2a [13], [28]. All tested adjuvants enhance the titer of anti-Aβ antibodies induced by (1–11)E2.

The (1–11)E2 antigen, with its intrinsic ability to induce an IgG1-biased immune response, thus lends itself as a promising candidate for the development of alum or squalene oil-in-water emulsion adjuvanted, or even unadjuvanted, anti-Aβ immunogens. *In vivo* experiments in mouse models of Alzheimer's Disease need to be performed to analyze the effects of immunization with (1–11)E2 on the levels of different Aβ species, and on cognitive endpoints.

## Supporting Information

Figure S1 **Titration curves of secondary anti-IgG1 and anti-IgG2a antibodies.** The graph shows the absorbance of ELISA wells coated with IgG1 (clone BAM-10) and IgG2a (clone NAB-228) antibodies and incubated respectively with secondary anti-mouse IgG1 (black symbols) or anti-mouse IgG2a (open symbols). The x-axis error bars report the percentage error in replicate measures of the concentration of primary antibody, the y-axis error bars report the standard deviation in the absorbance of replicate wells. At the same concentration of primary antibody, wells incubated with anti-mouse IgG1 and anti-mouse IgG2a secondary antibodies show no significant difference of absorbance.
(TIF)

Figure S2 **Isotype of anti-Aβ antibodies induced by LPS-free (1–11)E2 in individual mouse sera.** **panel a** The histograms show the measured light absorbance in ELISA wells coated with Aβ and incubated with the sera from five mice (ALF1, ALF2, ALF3, ALF4, ALF5) vaccinated with LPS-free (1–11)E2 in Alhydrogel 2%. Sera were collected at day 109, and tested at 1∶8000 dilution. Anti-Aβ antibodies were measured using secondary antibodies specific for IgG1 (open bars) or IgG2a (black bars). Histogram bars represent the mean absorbance of replicate wells, the error bars represent the standard deviation. IgG1 antibodies were more abundant than IgG2a in 4/5 mice. **panel b** The histograms show the measured light absorbance in ELISA wells coated with Aβ and incubated with the sera from four mice (SLF2, SLF3, SLF4, SLF5) vaccinated with LPS-free (1–11)E2 in AddaVax. Sera were collected at day 109, and tested at 1∶2000 dilution. Anti-Aβ antibodies were measured using secondary antibodies specific for IgG1 (open bars) or IgG2a (black bars). Histogram bars represent the mean absorbance of replicate wells, the error bars represent the standard deviation. IgG1 antibodies were more abundant than IgG2a in 4/4 mice. **panel c** The histograms show the measured light absorbance in ELISA wells coated with Aβ peptide Abeta(1–11) and incubated with the sera from five mice (PLF1, PLF2, PLF3, PLF4, PLF5), vaccinated with LPS-free (1–11)E2. Sera were collected at day 35, and tested at 1∶2000 dilution. Anti-Aβ antibodies were measured using secondary antibodies specific for IgG1 (open bars) or IgG2a (black bars). Histogram bars represent the mean absorbance of replicate wells, the error bars represent the standard deviation. IgG1 antibodies were more abundant than IgG2a in 4/5 mice. **panel d** The graph shows the anti-Aβ (1–11) titer measure on the sera of seven APP PSEN1 mice immunized with (1–11)E2 formulated in Alhydrogel 2%. Mice received 3 doses of adjuvanted vaccine, at day 0, 15 and 30. Sera were collected at day 50. Anti-Aβ antibodies were measured using secondary antibodies specific for IgG, IgG1 or IgG2a. Each dot represents a mouse. In all tested sera IgG1 antibodies were more abundant than IgG2a. IgG2a were under the limit of detection at the 1∶100 dilution in the sera of 5 out of 7 mice.
(TIF)

Figure S3 **Persistence of the anti-beta amyloid titer in individual mice.** The plot reports the anti-Aβ antibody titer at day 109 of B6C3/F1 mice immunized (day 0 and day 21) with LPS-free (1–11)E2, formulated in Alhydrogel 2% (diamonds), AddaVax (circles), or without adjuvant (triangles). Each symbol represents a mouse. In the adjuvanted groups, the anti-Aβ titer persisted above 1∶15000 in all individuals, wheras in 2/5 mice receiving the unadjuvanted vaccine the day 109 titer is below 1∶1000.
(TIF)

Figure S4 **Recognition of distinct Aβ species by sera from mice immunized with differently adjuvanted formulations of the LPS-free (1–11)E2 antigen.** Immuno-dot blot analysis conducted on 60 ng each of the indicated Aβ42 species (*M*, monomers; *O*, oligomers; *P*, protofibrils; *F*, fibrils) with 1∶140,000 (Alhydrogel 2%), 1∶80,000 (Addavax) and 1∶5000 (PBS) dilutions of pooled (day 77) sera from B6C3/F1 mice.
(TIF)
