# Supplementary figures and images for: Alum and Squalene-Oil-in-Water Emulsion Enhance the Titer and Avidity of Anti-Aβ Antibodies Induced by Multimeric Protein Antigen (1–11)E2, Preserving the Igg1-Skewed Isotype Distribution
Source: PLoS One. 2014 Jul 1;9(7):e101474. doi: 10.1371/journal.pone.0101474 (PMC4077797; doi:10.1371/journal.pone.0101474)

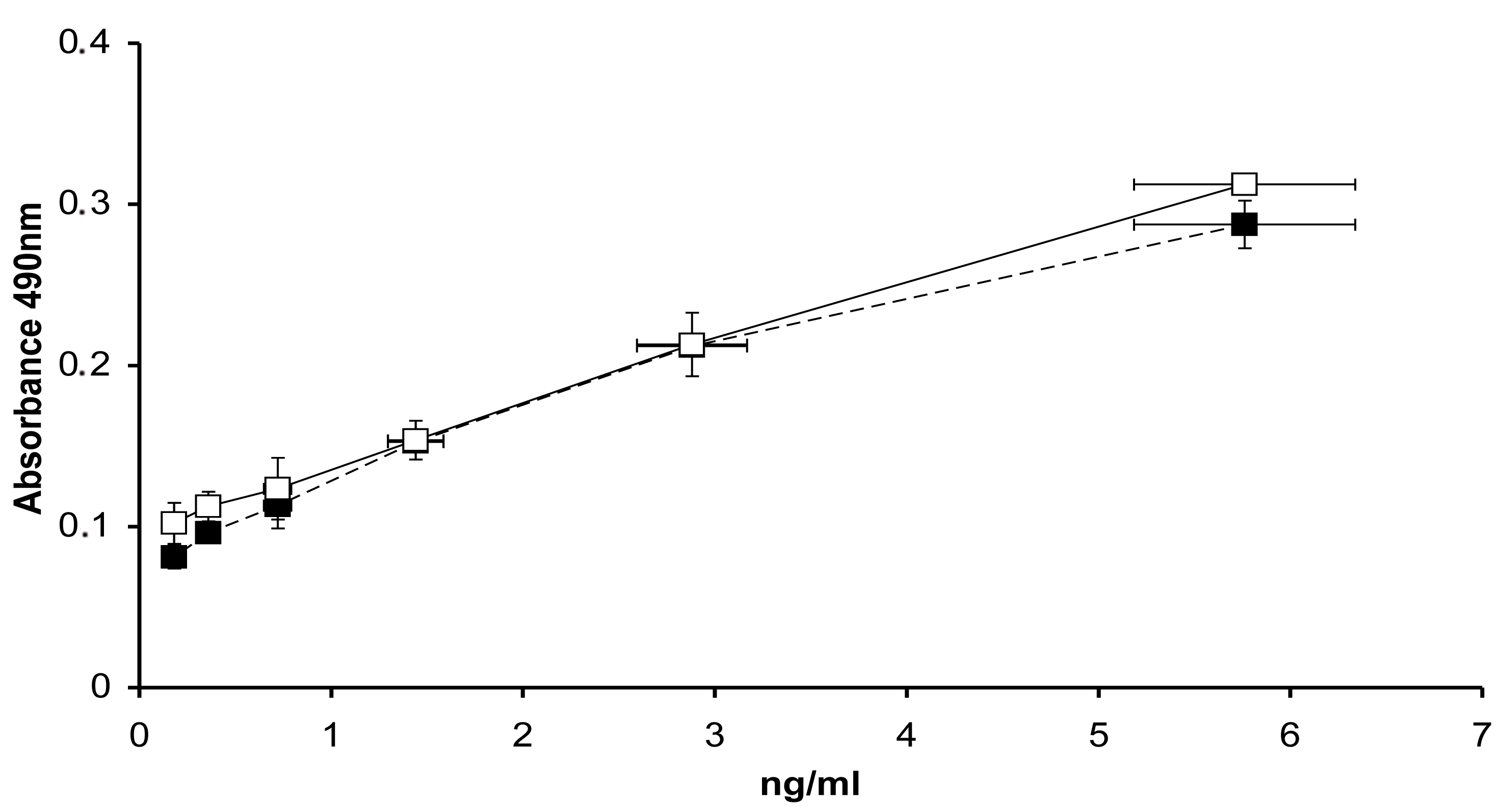

Supplement: Figure S1 — Titration curves of secondary anti-IgG1 and anti-IgG2a antibodies. The graph shows the absorbance of ELISA wells coated with IgG1 (clone BAM-10) and IgG2a (clone NAB-228) antibodies and incubated respectively with secondary anti-mouse IgG1 (black symbols) or anti-mouse IgG2a (open symbols). The x-axis error bars report the percentage error in replicate measures of the concentration of primary antibody, the y-axis error bars report the standard deviation in the absorbance of replicate wells. At the same concentration of primary antibody, wells incubated with anti-mouse IgG1 and anti-mouse IgG2a secondary antibodies show no significant difference of absorbance. (TIF) [file pone.0101474.s001.tif]

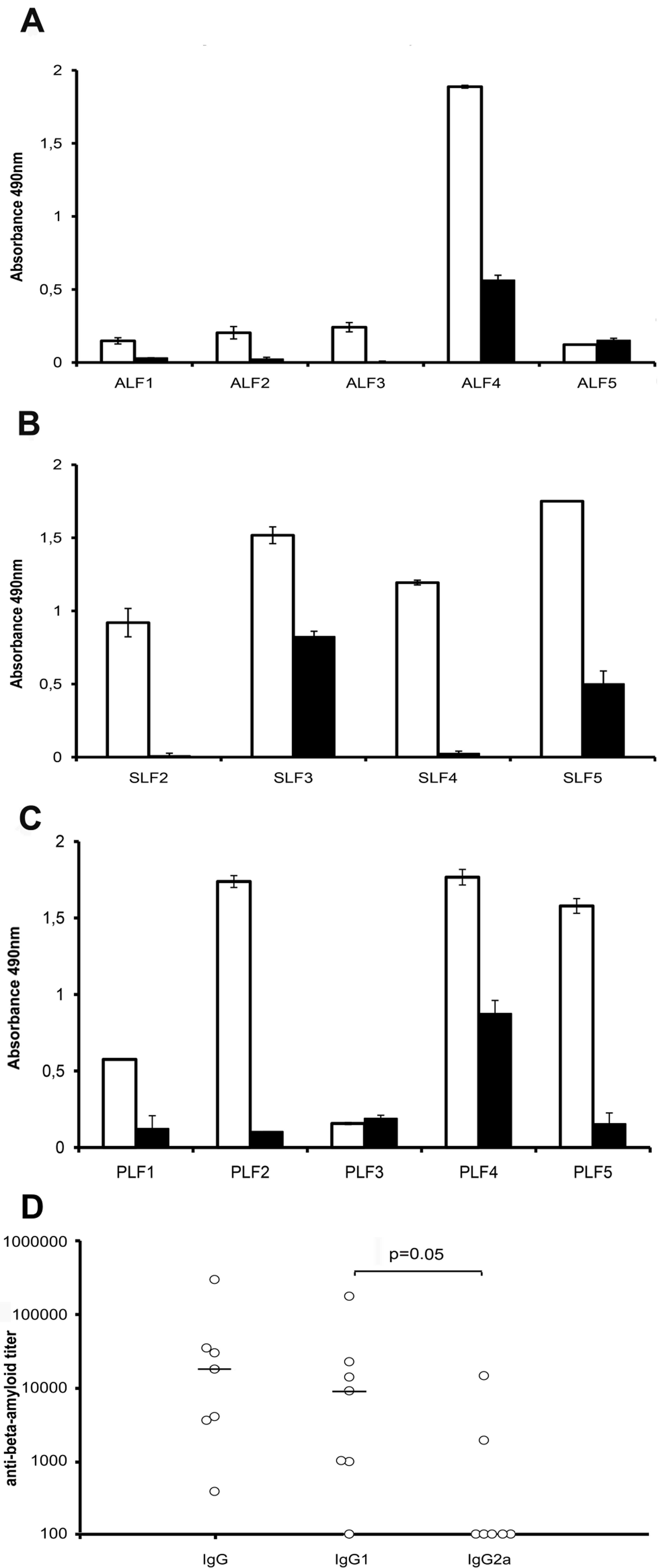

Supplement: Figure S2 — Isotype of anti-Aβ antibodies induced by LPS-free (1–11)E2 in individual mouse sera. panel a The histograms show the measured light absorbance in ELISA wells coated with Aβ and incubated with the sera from five mice (ALF1, ALF2, ALF3, ALF4, ALF5) vaccinated with LPS-free (1–11)E2 in Alhydrogel 2%. Sera were collected at day 109, and tested at 1∶8000 dilution. Anti-Aβ antibodies were measured using secondary antibodies specific for IgG1 (open bars) or IgG2a (black bars). Histogram bars represent the mean absorbance of replicate wells, the error bars represent the standard deviation. IgG1 antibodies were more abundant than IgG2a in 4/5 mice. panel b The histograms show the measured light absorbance in ELISA wells coated with Aβ and incubated with the sera from four mice (SLF2, SLF3, SLF4, SLF5) vaccinated with LPS-free (1–11)E2 in AddaVax. Sera were collected at day 109, and tested at 1∶2000 dilution. Anti-Aβ antibodies were measured using secondary antibodies specific for IgG1 (open bars) or IgG2a (black bars). Histogram bars represent the mean absorbance of replicate wells, the error bars represent the standard deviation. IgG1 antibodies were more abundant than IgG2a in 4/4 mice. panel c The histograms show the measured light absorbance in ELISA wells coated with Aβ peptide Abeta(1–11) and incubated with the sera from five mice (PLF1, PLF2, PLF3, PLF4, PLF5), vaccinated with LPS-free (1–11)E2. Sera were collected at day 35, and tested at 1∶2000 dilution. Anti-Aβ antibodies were measured using secondary antibodies specific for IgG1 (open bars) or IgG2a (black bars). Histogram bars represent the mean absorbance of replicate wells, the error bars represent the standard deviation. IgG1 antibodies were more abundant than IgG2a in 4/5 mice. panel d The graph shows the anti-Aβ (1–11) titer measure on the sera of seven APP PSEN1 mice immunized with (1–11)E2 formulated in Alhydrogel 2%. Mice received 3 doses of adjuvanted vaccine, at day 0, 15 and 30. Sera were collected at [file pone.0101474.s002.tif]

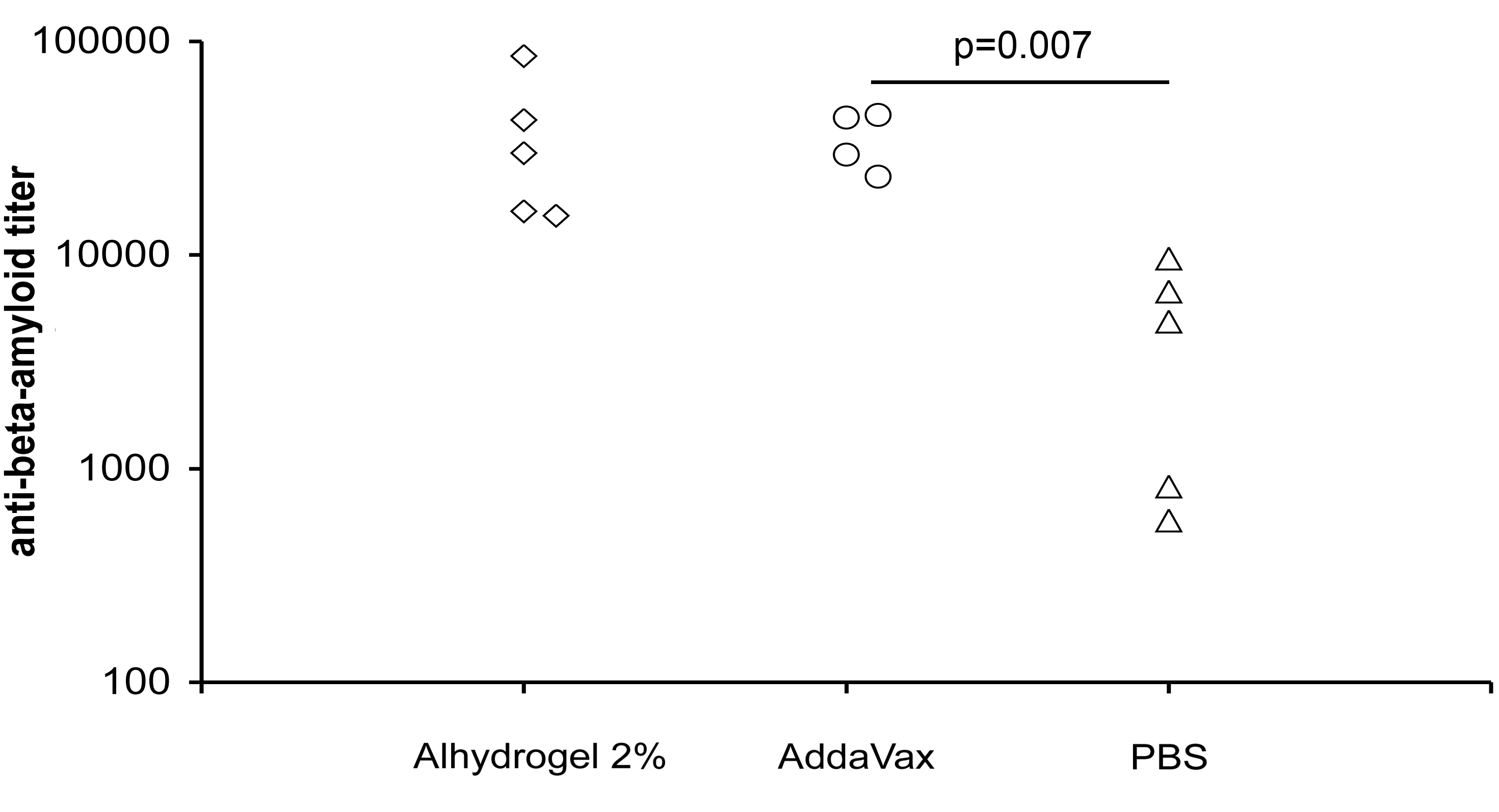

Supplement: Figure S3 — Persistence of the anti-beta amyloid titer in individual mice. The plot reports the anti-Aβ antibody titer at day 109 of B6C3/F1 mice immunized (day 0 and day 21) with LPS-free (1–11)E2, formulated in Alhydrogel 2% (diamonds), AddaVax (circles), or without adjuvant (triangles). Each symbol represents a mouse. In the adjuvanted groups, the anti-Aβ titer persisted above 1∶15000 in all individuals, wheras in 2/5 mice receiving the unadjuvanted vaccine the day 109 titer is below 1∶1000. (TIF) [file pone.0101474.s003.tif]

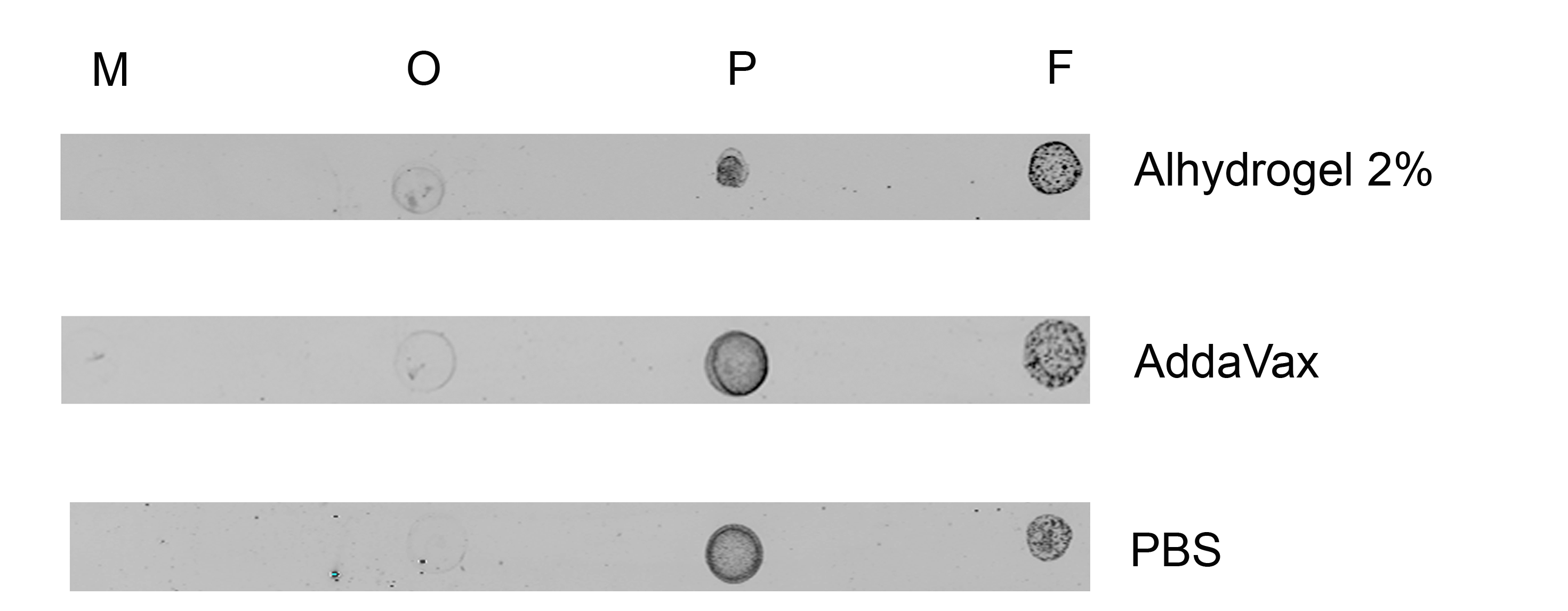

Supplement: Figure S4 — Recognition of distinct Aβ species by sera from mice immunized with differently adjuvanted formulations of the LPS-free (1–11)E2 antigen. Immuno-dot blot analysis conducted on 60 ng each of the indicated Aβ42 species (M, monomers; O, oligomers; P, protofibrils; F, fibrils) with 1∶140,000 (Alhydrogel 2%), 1∶80,000 (Addavax) and 1∶5000 (PBS) dilutions of pooled (day 77) sera from B6C3/F1 mice. (TIF) [file pone.0101474.s004.tif]
